# The Influence of Heat on Pediatric and Perinatal Health: Risks, Evidence, and Future Directions

**DOI:** 10.3390/jcm14041123

**Published:** 2025-02-10

**Authors:** Nicola Principi, Beatrice Rita Campana, Alberto Argentiero, Valentina Fainardi, Susanna Esposito

**Affiliations:** 1 Università degli Studi di Milano, 20122 Milan, Italy; nicola.principi@unimi.it; 2 Pediatric Clinic, Department of Medicine and Surgery, University of Parma, 43126 Parma, Italy; beatricerita.campana@unipr.it (B.R.C.); alberto.argentiero@unipr.it (A.A.); valentina.fainardi@unipr.it (V.F.)

**Keywords:** heat stress disorders, extreme health, climate change, global warming, pregnancy complications, preterm birth, pediatrics

## Abstract

Children, particularly infants and those with chronic conditions, are highly vulnerable to heat-induced health risks, similarly to the elderly. This narrative review synthesizes current evidence on the impact of heat exposure on pediatric and perinatal health. A systematic literature search was conducted using PubMed/MEDLINE and manual reference checks, focusing on studies from 2000 to 2024. Findings indicate that maternal heat exposure is associated with adverse pregnancy outcomes, including pre-eclampsia, gestational diabetes, hypertension, and increased hospital admissions. Additionally, prenatal heat stress correlates with preterm birth, low birth weight, birth defects, and stillbirth. In childhood, heat-related health consequences range from heatstroke and dehydration to renal impairment, respiratory diseases, and gastrointestinal infections. Psychosocial effects, including cognitive impairment, sleep disturbances, and mental health issues, have also been reported in school-age children and adolescents. Despite strong epidemiological evidence, critical knowledge gaps remain, including the exact temperature thresholds that increase disease risk and how these thresholds vary by age and underlying health conditions. Urgent public health measures are required to mitigate these risks, while further research is needed to define exposure–response relationships and effective interventions. Addressing the rising burden of heat-related pediatric illness is essential in the context of climate change and increasing global temperatures.

## 1. Introduction

Due to uncontrolled human activities over the last 50 years, primarily the emission of greenhouse gases from fossil fuel combustion, significant climate changes have occurred globally, leading to dramatic consequences for the entire world’s population [1]. The increase in the average global temperature is the most significant expression of climate change. It causes substantial clinical problems, induces significant changes in snow cover and sea ice, intensifies heavy rainfall, and alters habitat ranges for plants and animals. Every form of life on Earth has been, and continues to be, severely affected by the rise in temperature [1]. Unfortunately, long-term temperature observations have shown that the rate of this increase is accelerating. The average temperature recorded in 2014, the year with the highest temperature at that time, was surpassed by 2015, which was then exceeded by 2016 [2]. In 2023, the global temperature was the highest since records began in 1850, at 1.18 °C above the 20th-century average of 13.9 °C [3]. The impact of temperature increases on health, the environment, and the economy has already been severe [4], and without effective measures to address the causes of climate change, these impacts will continue to worsen.

Alongside the elderly, children, especially the youngest and those with severe chronic underlying diseases, are particularly susceptible to heat-induced adverse health outcomes. Several factors explain why children are at increased risk of severe clinical problems when exposed to increased temperatures or prolonged heatwaves [1,2]. These include the limitations of their thermoregulatory systems, a higher risk of dehydration, and the inability to implement common measures to reduce ambient temperature.

This narrative review discusses the most important heat-related medical problems that can occur in children. A comprehensive literature review was conducted to identify and synthesize relevant studies on the impact of heat exposure on pediatric and perinatal health. The search strategy included a systematic query of the PubMed/MEDLINE database, using the following keywords: “heat” OR “increased temperature” AND “pregnancy” OR “fetus” OR “neonate” OR “newborn” OR “infant” OR “children” OR “adolescents”. The search covered studies published from 2000 to February 2024 to ensure the inclusion of the most recent and relevant findings. Additional relevant literature was identified through manual searches of reference lists from key articles, systematic reviews, and meta-analyses. This review focused on peer-reviewed studies published in English, prioritizing original research, systematic reviews, and large-scale epidemiological studies with robust methodologies.

To ensure the selection of high-quality and relevant studies, inclusion criteria required that articles met the following criteria:

1. Provided empirical data on the relationship between heat exposure and health outcomes in fetuses, neonates, children, or adolescents.
2. Included well-defined study populations and exposure measures (e.g., temperature thresholds, duration of exposure, and health outcomes).
3. Reported statistical analyses, such as risk estimates (e.g., odds ratios, relative risks, hazard ratios) and confidence intervals.

Studies with small sample sizes, weak methodologies, or insufficient statistical analysis were excluded unless they provided unique insights or were part of broader systematic reviews. Data were extracted and synthesized according to thematic categories, including perinatal outcomes (preterm birth, low birth weight, stillbirth), pediatric morbidity (heatstroke, dehydration, infections, renal impairment), and psychosocial impacts. The final selection of articles was based on originality, methodological rigor, and relevance to the overarching scope of this review. The initial search generated 2703 studies, from which 813 duplicates were removed. Thus, 1890 studies were screened in the title and abstract phase. Of the 517 studies assessed in full text review, 359 were excluded, mainly due to lack of a pediatric population and lack of specificity for heatwaves or extreme heat events. Ultimately, 158 studies met criteria to be included in the review.

## 2. Heat Exposure During Pregnancy

Exposure to heat can be detrimental for pregnant women. High seasonal temperatures have been linked to several pregnancy complications, including pre-eclampsia [5], gestational diabetes [6], and an increased number of emergency hospital admissions during pregnancy [7]. Furthermore, heat-exposed mothers are more likely to experience hypertension and have longer hospital stays at childbirth [7].

Even more significant damage can occur in the fetus. Accumulating evidence suggests a positive association between prenatal heat exposure and the occurrence of premature birth, lower birth weight, increased prevalence of birth defects, and stillbirth [8]. Although the exact reasons for these complications are not definitively established, several factors, such as biological effects, behavioral effects, and income effects, may play a role, with biological effects being the most significant [8]. Increased air temperatures cause blood to be diverted to the skin to enhance heat loss, reducing blood flow to the placenta and limiting oxygen and nutrient supply to the fetus [8]. This can result in the previously reported fetal complications [9]. Additionally, dehydration can trigger the release of prostaglandin and oxytocin, potentially leading to premature labor [10]. Heat stress also prompts the production of heat-shock proteins, which can damage the placental structure and function, further increasing fetal damage [11]. Behavioral and income effects may also complicate pregnancy. For example, increased temperatures can reduce appetite and alter food selection, causing nutritional problems for the fetus [12]. In rural settings, temperature increases can impact household resources and nutrition for pregnant women by affecting crop yields, thereby influencing fetal health [13].

A large number of studies strongly support the potential association between heat exposure during pregnancy and fetal damage. Unfortunately, these studies are very heterogeneous, and it is not precisely defined which period of maternal sensitivity is critical, what minimal temperature is associated with increased risk of fetal problems, and how long exposure to increased heat must last to cause fetal damage. Moreover, it is not known whether certain women have a higher sensitivity to temperature increases, although it has been suggested that extreme child-bearing ages, lower socioeconomic positions, lower educational attainment, or living in low-resource settings may be factors that favor fetal damage. Despite these limitations, some studies involving a large number of pregnant women and some recently published meta-analyses of available data allow for drawing some interesting, although not definitive, conclusions.

### 2.1. Risk of Prematurity

Regarding prematurity, a study conducted in China involving 210,798 singleton live births found that exposure to extreme heat (>95th percentile of mean outdoor temperature) during early, late, and the entire pregnancy was associated with an increased risk of both medically indicated preterm birth (hazard ratio [HR] 1.63, 95% confidence intervals [CI] 1.19–2.22) and spontaneous preterm birth (HR 1.50, 95% CI 1.11–2.02) [14]. Furthermore, a meta-analysis of 70 studies from 27 countries confirmed that both acute and chronic exposure to increased temperature played a role in preterm births. Increased numbers of preterm babies were observed when pregnant women were exposed to increased temperatures one month preconception, the month of conception, the first or second trimester of pregnancy, and the last week before birth [15]. Moreover, it was shown that the risk was higher with higher temperatures, with each additional degree Fahrenheit (0.56 °C) being associated with a 5% increased risk. A similar trend was reported in a recent study that examined changes in daily rates of preterm and early-term births after heatwaves in a 25-year nationwide evaluation [16]. Among 53,154,816 eligible births in the 50 US metropolitan statistical areas from 1993 to 2017, 2,153,609 preterm births and 5,795,313 early-term births occurring in the warm season were analyzed. It was found that heatwaves were positively associated with daily rates of preterm and early-term births, showing a dose–response association with heatwave duration and temperatures and stronger associations in the more acute 4-day window [16]. After four consecutive days of mean temperatures exceeding the local 97.5th percentile, the rate ratio for preterm birth was 1.02 (95% CI, 1.00–1.03), and the rate ratio for early-term birth was 1.01 (95% CI, 1.01–1.02). For the same exposure, among pregnant women who were 29 years of age or younger, had a high school education or less, and belonged to a racial or ethnic minority group, the rate ratios were 1.04 (95% CI, 1.02–1.06) for preterm birth and 1.03 (95% CI, 1.02–1.05) for early-term birth [16]. Despite their limited clinical significance, these differences underline the need for further studies in this regard, especially in low-income countries.

### 2.2. Risk of Low Birth Weight

As for low birth weight, published studies indicate that this complication of exposure to increased temperature during pregnancy is relatively common, albeit with marginal clinical relevance [15,17]. Dalugada et al., after evaluating 75 studies published between 2015 and 2020, reported that low birth weight was the second most common heat-associated adverse fetal event after prematurity [17]. An attempt to quantify the risk was made by Chernish et al., who reported that the low birth weight rate was 9% higher during periods with hotter than usual temperatures [15]. However, the reduction in weight was only a few grams, varying from 1 to 26 g. More significant impacts on birth weight were evidenced by Ranciere et al., who found that mothers who were pregnant during the 2003 French heatwave were more likely to have a term small-for-gestational-age (SGA) baby (adjusted odds ratio [aOR] = 2.70, 95% CI: 1.38–5.28) compared to mothers who did not experience a heatwave during pregnancy [18]. The association was stronger when the heatwave occurred during the first trimester (aOR = 4.18, 95% CI 1.69–10.35). Primiparous women were identified as more vulnerable than multiparous women. The average ambient temperature and the air quality index explained about 36% and 56% of the association between experiencing a heatwave during the first trimester and term SGA, respectively [18].

### 2.3. Risk of Birth Defects

Regarding birth defects, most studies have reported that cardiovascular abnormalities are the most common birth defects following heat exposure during pregnancy, although there are exceptions [19]. The risk of congenital heart defects may be up to three times higher, especially with higher temperatures and when heat exposure occurs during the first weeks of pregnancy when the cardiac structure is being defined [20]. These conclusions are exemplified by data from a population-based case-control study by Lin et al. in the USA involving data from eight research centers, 5848 congenital heart defect cases, and 5742 controls. The study showed that 3–11 days of extreme heat events during summer and spring were significantly associated with ventricular septal defects (odds ratios [ORs] ranged from 2.17 to 3.24) and conotruncal defects (ORs ranged from 1.23 to 1.78) [21]. Furthermore, it was reported that three to five days of high temperatures (daily maximum temperature >90th percentile) at weeks 5 to 10 of gestation were associated with higher rates of ventricular septal defects (ORs ranged from 2.17 to 2.57, all *p* < 0.05). Although less common, other birth defects have been associated with heat exposure during pregnancy, including hypospadias, congenital cataracts, renal agenesis/hypoplasia, spina bifida, and craniofacial defects [21]. However, the reliability of these findings remains debated, as results are conflicting. Some studies have found no certain association between heat exposure and malformation development [20]. Other studies, such as those by Van Zutphen et al. and Auger et al., have found specific malformations related to heat exposure [22,23]. Van Zutphen et al. conducted a study in the USA involving 6422 children with birth defects and 59,328 controls, finding that a 5-degree increase in the mean daily minimum universal apparent temperature was significantly associated with congenital cataracts (OR 1.51, 95% CI: 1.14–1.99) [22]. Auger et al. conducted a study in Canada, retrospectively enrolling about 900,000 live births over 24 years, and found that exposure to maximum temperatures above 30 °C during day 5 (OR 1.56, 95% CI 1.04–2.35) and day 6 (OR 1.49, 95% CI 1.00–2.21) of week 4 of gestation was associated with a neural tube defect [23].

### 2.4. Risk of Stillbirth

Recent reviews and meta-analyses have shown a high risk of stillbirth following maternal heat exposure [15,24]. Chersich et al. examined eight studies and found that stillbirths increased by 1.05-fold (95% CI, 1.01–1.08) per 1 °C rise in temperature, by 1.24-fold (95% CI, 1.12–1.36) when the heat increase occurred in the last week of pregnancy, and by 3.39-fold (95% CI, 2.33–4.96) when temperature effects were examined over a trimester or the entire pregnancy period [15]. Sexton et al. confirmed that the risk of stillbirth is present throughout pregnancy but is greater in late pregnancy [24]. These authors reported that the risk tends to increase above 23.4 °C, with the highest risk above 29.4 °C. Data from developing countries, where heatwaves are common, have provided more evidence. In Ghana, data on 5,961,328 births indicated that the risk of stillbirth increased with the duration of heat exposure and was stronger for exposure to 30.8 °C for the entire pregnancy, with an 18% (95% CI 2–36) greater risk [25]. However, two studies from Iran reported differing results. One study found that exposure to 46.4 °C during the last two weeks of pregnancy was associated with a doubled risk of stillbirth compared to exposure to 38.0 °C [26]. In contrast, another study using different methods to assess temperature variations and heat exposure found no increase in risk with rising temperatures [27]. This indicates that the method used to evaluate exposure to high temperatures is critical for identifying the potential effects of heat on pregnancy outcomes.

### 2.5. Risk of Postnatal Clinical Problems

Exposure to extreme heat during pregnancy may impact newborn health and have effects later in life [7,26,27,28,29]. Newborns of mothers exposed to extreme heat were more frequently dehydrated (31%) and had a higher risk of readmission to the hospital in the first year of life (3.4%) [7]. Cil and Cameron reported that a heatwave during the third trimester was associated with a 3.5% increase in the number of cases with fetal distress, meconium aspiration, or neonatal ventilator use [28]. Kakkad et al. showed a strong relationship between heat and NICU admissions, finding that temperatures exceeding 42 °C led to a 43% increase in heat-related NICU admissions for every 1 °C increase (95% CI 9.2–88%) [29]. Long-term consequences were also noted in a study conducted in China, which found that adults born to mothers who experienced an additional high-temperature day during pregnancy had some mild, though not insignificant, physical and academic problems [13]. On average, the risk of illiteracy increased by 0.18%, word test scores were lower by 0.48%, and final height was reduced by 0.02 cm [13]. Predictions suggest that by the end of the 21st century, a further reduction in years of education (0.14–0.54) and height (0.21–0.84 cm) might occur due to continued temperature increases [13]. Additionally, an extra day with mean temperatures above 32 °C in utero was associated with a 0.1% reduction in adult annual earnings at age 30 [30]. Table 1 summarizes the consequences of heat exposure during pregnancy.

## 3. Physical Effects of Heat Exposure in Infants and Children

For years, children were considered significantly more vulnerable than adults to increased outdoor temperatures due to less efficient thermoregulation [31]. This conclusion was initially based on evidence that in response to physical activity in the heat, children, especially the youngest, exhibited higher core temperatures, higher heart rates, and lower sweat rates. Definitive support for this conclusion came from several studies on the mechanisms that maintain thermal homeostasis [31]. These studies found that children’s sweat rates were about half those of adults, leading to a poor ability to reduce body temperature through evaporative loss. Additionally, children’s higher body surface area to body mass ratio exposed them to a higher risk of body heat gain when air temperature exceeded skin temperature [31]. Finally, despite higher skin blood flow in children compared to adults, their lower cardiac output limited heat loss [31].

Recent revisions in pediatric thermoregulation have concluded that in mild to moderate environmental conditions, children’s ability to maintain body temperature within the normal range does not differ from that of adults, although children use different thermoregulation strategies [31]. In such conditions, children’s higher body surface area to body mass ratio becomes advantageous, allowing more efficient heat dissipation through non-evaporative means. This compensates for their lower sweat production and limits fluid loss. When cardiac output is measured relative to surface area, it is similar to that of adults, and, combined with higher skin blood flow, it ensures greater non-evaporative heat loss [31].

However, in very hot environments, especially during prolonged heatwaves, children can experience significant clinical problems as non-evaporative heat dissipation declines [29]. Infants are at the greatest risk due to greater energy expenditure, higher sweating thresholds, smaller blood volumes, and higher heart rates, which affect the cardiovascular response to extreme temperatures [29,32]. Older children also face challenges, as they take longer to acclimatize to higher temperatures due to the time required to produce a significant amount of sweat [31]. Toddlers and preschool children are particularly vulnerable because they cannot enact behavioral modifications in response to heat, often remaining in thermally threatening environments when outdoor temperatures rise [33]. They cannot control their clothing or perceive thermal discomfort accurately, often staying outdoors under the sun for extended periods before seeking shade or air-conditioned environments [34]. Furthermore, children may be unable to effectively communicate their thermal status or thirst to adults, relying entirely on caregivers to ensure their thermal comfort [35]. Unfortunately, several studies have shown that parents and caregivers often fail to accurately assess the thermal state of children [36,37]. Consequently, children’s water intake is typically lower than recommended when exposed to hot temperatures, increasing their risk of dehydration and severe clinical manifestations compared to adults [38,39,40]. The risk of heatstroke and dehydration, as well as the impact of heat on pediatric patients with chronic diseases, are priority topics for further studies.

### Risk of Morbidity and Mortality

The association between heatwaves or periods of hot temperatures and increased pediatric morbidity and mortality has been repeatedly demonstrated, especially among younger children, those with severe chronic underlying diseases, and those living in low- and middle-income settings [41,42,43,44,45]. Unfortunately, few studies have evaluated cause-specific mortality, and results have often been conflicting. In California, it was found that during hot days, infant mortality was primarily due to problems originating in the perinatal period [32]. In Spain, no specific cause was identified [46]. Attempts to correlate heat exposure with sudden infant death syndrome (SIDS) have also yielded inconsistent results. In Korea, a study showed that the overall hazard ratio of infant mortality from SIDS for a 1 °C increase during the month before death was 1.50 (95% CI, 1.35–1.66) [42]. Similar findings were reported in the USA [47] and Canada [41], suggesting a potential association further confirmed by the evidence that the temperature–SIDS association was most robust among cities with lower air conditioning prevalence. However, another study in the USA [32] and a study in Austria [48] found no increased risk of SIDS during hot periods. Regarding morbidity, no specific clinical manifestation has been positively associated with hot temperatures except hand, foot, and mouth disease [49]. Most studies report that during hot periods, there is a greater than expected number of children admitted to hospitals. In New York, it was found that children aged 0–4 years had an increased risk of emergency department visits with increased Tmax on lag days 0, 1, and 3, with the strongest association on lag day 0, where an increase in Tmax of 13 °F conferred an excess risk of 2.6% (95% CI 2.2–3.0). Significant positive associations were observed for same-day exposures for 1- to 4-year-olds, while children less than 1 year of age showed a significant positive association with Tmax only on lag day 3 [50].

In a subtropical city in Australia, it was shown that heatwave intensity was critical in conditioning the risk of hospital admission. Temperatures in the ≥90th or ≥95th percentile of the mean temperature across the study period had no influence on hospital admission rates. However, when heatwave intensity increased to the ≥97th percentile, hospital admissions significantly increased (relative risk [RR] 1.05, 95% CI 1.01–1.10) [51].

Clinical manifestations secondary to heat exposure can vary significantly according to several factors, including child characteristics, degree of temperature increase, and length of exposure (Table 2) [52,53]. In the most severe cases, heatstroke and death can occur, such as in infants and toddlers left in hot cars [52] or who collapse during sports activities in hot weather [53]. Most exertional heat illness cases in preschool athletes are diagnosed during preseason football practice due to high ambient temperatures, poor physical fitness, limited acclimatization, and repeated training sessions [54].

Less severe cases commonly report heat-related skin injuries, fever, renal impairment, respiratory diseases, and infections [55]. Studies conducted during hot periods in England and Australia reported significant increases in calls for fever among children aged 0–4 years and younger than 6 years, respectively [56,57]. Renal failure and other fluid, electrolyte, and acid–base balance disorders, primarily due to dehydration, are common [58,59]. A study in Brazil involving 2,726,886 hospitalizations for renal diseases found that 7.4% (95% CI 5.2–9.6%) of them could be attributed to increased temperatures, accounting for 202,093 (95% CI 141,554–260,594) cases [58]. Every 1 °C increase in the daily mean temperature increased the risk of hospitalization for renal diseases over lag 0–7 days by 0.9% (RR 1.009, 95% CI 1.008–1.010), with children aged 0–4 years among the highest risk groups [56].

Hot temperatures have also been associated with an increase in bacterial diarrheal diseases, as high temperatures favor the survival of enterotoxigenic bacteria in contaminated food and behaviors like increased water consumption and lax hygiene, which can promote diarrheal transmission [58]. An epidemiological study involving children under six years old found that the maximum daily temperature was a significant risk factor for gastroenteritis (*p* = 0.007), with a regression coefficient of 0.10 [57].

## 4. Psychosocial Problems

The possible association between higher-than-usual outdoor temperatures and the development of acute mental health problems was documented as early as the 1970s [60]. However, it is only since 2003 that this issue has gained widespread interest and been extensively studied [61,62,63,64]. Recent studies have confirmed this association, showing that increased ambient temperature correlates with a significant rise in emergency department visits for mental illness, suicides, and self-reported days of poor mental health [61,62,63]. Individuals with pre-existing mental health issues are at the greatest risk. In particular, individuals with conditions like anxiety disorders, depression, or bipolar disorder may experience intensified symptoms during heatwaves, making it crucial to manage their mental health effectively. Several factors may explain the relationship between heat and mental illness [63,64,65,66]. Heat exposure can lower positive emotions and increase negative emotions and fatigue, causing negative mental states, including distress and exhaustion [64]. Heat disrupts sleep, which is extremely sensitive to environmental characteristics, leading to difficulty falling asleep, poor sleep quality, and fewer sleep hours [65]. This disruption can contribute to the occurrence of major mental health disorders [65]. Finally, heat exposure is associated with increased irritability, aggression, and violence, which, in turn, can lead to mental health problems [66].

As with the impact of heat on pregnancy, studies on heat and mental health are highly heterogeneous, making it difficult to draw definitive conclusions about the characteristics and prognosis of heat-related mental health issues [67]. Thompson et al. conducted a systematic review, incorporating a qualitative narrative synthesis and quantitative meta-analysis of studies published up to April 2022, selecting 144 epidemiological, observational studies involving humans of all ages, primarily adults. Due to high heterogeneity, the authors concluded that much of the evidence was of low certainty [67]. The need for further studies to define the temperatures and durations of exposure that may cause mental health problems, and whether different heat increases cause different mental health issues, was emphasized. Only 19 studies were suitable for meta-analysis, but even these had significant methodological limitations, leading to debatable conclusions [67]. The meta-analysis revealed that hospital attendance or admission for mental illness was significantly more common during heatwaves (defined as daily maximum temperatures of at least 35 °C for at least 3 days) than in non-heatwave periods (relative risk [RR] 9.7%, 95% CI 7.6–11.9, *p* < 0.001) [67]. Additionally, the risk ratio of hospitalization was slightly higher (RR 1.02, 95% CI 1.01–1.03, *p* = 0.006) when daily mean temperatures were at or above the 99th percentile compared to the 50th percentile [65]. An increase in the incidence of suicide by 1.5% (95% CI 0.8–2.2, *p* < 0.001) was found to be associated with a 1 °C increase in the mean monthly temperature. Similar effects were observed with a 1 °C increase in the mean daily temperature (incidence increase of 1.7%, 95% CI 0.3–3.0, *p* = 0.014) [67].

Evaluating the impact of heat on the mental health of children and adolescents remains challenging due to the limited number of studies. This makes it difficult to determine whether, when, and how younger individuals may experience mental health problems due to increased outdoor temperatures [68,69,70,71,72,73,74]. Moreover, no information is available regarding toddlers and preschool-aged children, a significant limitation given the high vulnerability of young people to mental health disorders [68] and the need for early intervention in children with pre-existing mental health issues to prevent further deterioration [69]. Initial studies have reported conflicting results. For example, Basu et al. [70] and Wang et al. [71] found that younger individuals were more sensitive to heat exposure than adults, while Chan et al. [72] and Trang et al. [73] reported the opposite. More information can be gleaned from a recent case-crossover study analyzing emergency department visits and hospital encounters for mental health disorders during the warm-season months (June–August) in New York City from 2005 to 2011 [74]. The study evaluated 82,982 mental-health-related encounters for individuals aged 6 to 25 years, including 6268 children aged 6–11 years, 22,206 adolescents aged 12–17 years, and 54,508 young adults (18–25 years) [74]. Elevated temperatures (defined as the 95th percentile of warm-season temperatures from 2005 to 2011) were associated with increased mental-health-related healthcare encounters in all groups. The association was stronger for children (OR 1.28, 95% CI 1.13–1.46) and adolescents (OR 1.17, 95% CI 1.09–1.25) but also evident in young adults (OR 1.09, 95% CI 1.04–1.15) [74]. Mental health problems differed by age, with reaction disorders more common among children, anxiety and bipolar disorders more common among adolescents, and psychosis and reaction disorders more common among young adults [74].

Additionally, exposure to increased temperatures during the learning period can persistently disrupt the learning process [75]. Park et al. demonstrated that thermal conditions globally influence cumulative learning, partly explaining the differences in educational achievement between countries based on the number of hot days during the summer [75]. In the USA, schools in the southern States performed worse than those in the northern States. Hotter temperatures during the year prior negatively affected student learning between the third and eighth grades, particularly in mathematics and among already disadvantaged students. Each additional day with a temperature of 26.7 °C or hotter reduced achievement only by approximately 0.04% of a standard deviation (95% CI −0.07–0.01, *p* = 0.071) [75]. However, when the analysis of the effect of heat exposure was concentrated on school days, it was found that each additional hot school day lowered achievement by 0.07% of a standard deviation (95% CI −0.12–−0.02, *p* = 0.01), whereas heat on non-school days had no effect (95% CI −0.04–0.06, *p* = 0.551) [75].

In conclusion, the impact of heat on the psychological and mental health of children and adolescents is an area requiring comprehensive exploration. Symptoms, such as irritability, anxiety, and decreased cognitive function, can significantly hinder their daily lives and long-term well-being. Current research provides a foundational understanding, yet many aspects remain underexplored. Studies must delve deeper into the physiological and psychological mechanisms behind heat-related stress, examining variables like socioeconomic status, geographic location, and access to cooling resources. Additionally, interventions and preventative measures should be developed and tested to mitigate these effects, thus ensuring that young people can maintain mental resilience in the face of rising temperatures. By prioritizing this research, we can better safeguard the mental health of future generations, fostering a society that is both aware of and equipped to handle the challenges posed by a warming world.

## 5. Prevention of Heat-Related Problems: Present and Future

Several plans to prevent and control heat-related clinical, social, and economic problems have been prepared in many countries, especially those with the highest incomes. According to WHO and the World Meteorological Organization suggestions [76], heat warning systems were developed, including several critical components designed to monitor, predict, and communicate the risks associated with heatwaves. These components include meteorological monitoring and prediction, health surveillance, risk communication, and response planning and coordination. The foundation of a heat warning system is accurate meteorological data. Advanced forecasting models and real-time monitoring are essential for predicting the onset, duration, and intensity of heatwaves. Collaboration with national meteorological agencies can enhance the accuracy and reliability of forecasts. Health surveillance involves tracking heat-related illnesses and deaths to identify trends and vulnerable populations. These data can inform public health strategies and resource allocation. Partnerships with hospitals, clinics, and emergency services are vital for timely data collection and analysis. Clear and effective communication is crucial for raising public awareness and ensuring appropriate responses to heat warnings. Communication strategies should include the following. (a) Public alerts: utilizing multiple channels, such as television, radio, social media, and mobile alerts, to disseminate heat warnings and safety advice. (b) Educational campaigns: providing information on recognizing the signs of heat-related illnesses and preventive measures, such as staying hydrated and avoiding strenuous activities during peak heat periods. (c) Community outreach: engaging community leaders and organizations to reach vulnerable populations and provide targeted support. A coordinated response plan ensures that all relevant agencies and stakeholders work together efficiently during heatwaves. Key elements of response planning include (a) cooling centers, i.e., establishing air-conditioned public spaces where individuals can seek relief from the heat; (b) emergency services, i.e., ensuring that emergency medical services are prepared to handle an influx of heat-related cases; and (c) resource allocation, i.e., distributing resources, such as water, fans, and cooling devices, to those in need, particularly vulnerable individuals.

Examining successful heat warning systems worldwide can provide valuable insights into best practices and lessons learned. Here are two notable examples. (a) The United States: the National Weather Service (NWS) [77] operates a comprehensive heat warning system, issuing heat advisories and warnings based on specific criteria, such as temperature and humidity levels. The NWS collaborates with public health agencies to disseminate information and provides detailed guidelines on heat safety. (b) France: the National Heatwave Plan [78], following the deadly heatwave of 2003, was implemented in France as a national heatwave plan, which includes a multi-tiered warning system, public awareness campaigns, and the establishment of cooling centers. The plan also involves regular evaluations and updates to improve its effectiveness.

While significant progress has been made in developing heat warning systems, several challenges remain. These include the following. (a) Climate change, i.e., the increasing frequency and intensity of heatwaves, which necessitates continuous adaptation and improvement of warning systems. (b) Data integration, i.e., integrating meteorological data with health surveillance systems, which can be complex but is essential for a comprehensive understanding of heatwaves’ impacts. (c) Equity, i.e., ensuring that all population groups, particularly the most vulnerable, receive timely and effective heat warnings and support.

Future directions for heat warning systems should focus on leveraging technology, enhancing cross-sector collaboration, and fostering community resilience. Innovations, such as artificial intelligence and machine learning, can improve predictive capabilities, while partnerships between government agencies, non-profits, and private sector entities can strengthen response efforts.

## 6. Conclusions

UNICEF estimates that 559 million children are currently exposed to high heatwave frequencies, a number projected to rise to 2.02 billion globally by 2050 [79]. The evidence reviewed highlights that extreme heat exposure poses significant health risks to children of all ages, with the most severe consequences observed in fetuses, neonates, and infants. However, school-age children and adolescents are also vulnerable to heat-induced physical and psychosocial effects, including cognitive impairments and mental health disorders. Despite growing concern, major knowledge gaps hinder the development of effective prevention and mitigation strategies. Critical uncertainties remain regarding threshold temperatures for specific pediatric health risks, age-dependent vulnerability, and the comparative impact of prolonged versus acute heat exposure. Furthermore, regional differences in climate conditions, socioeconomic factors, and healthcare access complicate the assessment of global risk patterns. Addressing these gaps is essential for formulating evidence-based public health policies. Urgent action is needed to implement preventive strategies, including improved maternal and pediatric heat adaptation measures, urban cooling initiatives, and early warning systems. While further research is crucial to refine exposure–risk relationships, immediate efforts must focus on mitigating the adverse health effects of rising temperatures in children, ensuring a proactive response to the escalating threats posed by climate change.

## Figures and Tables

**Table 1:** Potential consequences of heat exposure during pregnancy.

Subject	Consequence
Woman during pregnancy	Pre-eclampsia
	Gestational diabetes
	Emergency hospital admissions
Woman at birth	Hypertension
	Longer hospitalization
Fetus	Premature birth
	Low birth weight
	Birth defects
	Stillbirth

**Table 2:** Main physical manifestations secondary to heat exposure in childhood.

Severity	Clinical Manifestation
Severe cases	Heatstroke
	Death
	Renal failure
Mild to moderate cases	Gastroenteritis
	Mosquito-vectored arboviral diseases
	Respiratory diseases
	Fever
	Heat-related skin injuries

## Data Availability

Not applicable.
